# Continuation of immunosuppressive treatment may be necessary in IgA nephropathy patients with remission of proteinuria: Evaluation by repeat renal biopsy

**DOI:** 10.3892/etm.2013.1467

**Published:** 2013-12-31

**Authors:** MIAN-NA LUO, CUI-WEI YAO, BI-HUA XU, YONG-ZHI XU, WEI JING LIU, YONG-MIN FENG, JING-LI TAO, HUA-FENG LIU

**Affiliations:** Institute of Nephrology, Guangdong Medical College, Zhanjiang, Guangdong 524001, P.R. China

**Keywords:** course of treatment, immunoglobulin A nephropathy, immunosuppressive treatment, proteinuria, renal pathology

## Abstract

The present study aimed to evaluate the effects of an individualized, low-dose multi-drug immunosuppressive regimen for the treatment of immunoglobulin A nephropathy (IgAN). A preliminary investigation of the course of IgAN following immunosuppressive treatment was conducted based on repeat renal biopsies. Clinical and pathological data of 17 patients with IgAN who received repeat renal biopsies were analyzed retrospectively. In addition to basic treatment, 16 patients regularly received an individualized low-dose immunosuppressive regimen according to their clinical manifestations and pathological patterns following the first biopsy. Clinical parameters, including 24-h urinary protein excretion and levels of serum albumin, uric acid and total cholesterol were collected. Glomerular deposits of IgA and C3, as well as the activity and chronicity indexes of renal lesions were evaluated by semi-quantitative methods. The 24-h urinary protein excretion of the patients decreased significantly from the first biopsy (2.53±2.17 g/day) to the repeated biopsy (0.26±0.55 g/day) (P<0.001). Deposits of IgA and C3 in the glomerulus were persistent, but were reduced in quantity at the second biopsy. Although active renal lesions were observed in the majority of patients, the activity index decreased significantly from 3.18±1.33 prior to therapy to 2.47±0.80 following therapy (P<0.05), while the chronicity index did not change significantly (2.59±2.00 versus 2.76±1.89, respectively). The individualized, low-dose multi-drug immunosuppressive regimen used in the present study significantly minimized proteinuria, stabilized renal function and alleviated histological lesions in patients with IgAN without causing overt adverse effects during the short-term follow-up. In addition to proteinuria, renal pathological changes should be appraised when considering the withdrawal of immunosuppressants from IgAN treatment.

## Introduction

Immunoglobulin A nephropathy (IgAN), an immune complex-mediated glomerulonephritis, is the most common form of primary glomerulonephritis worldwide and is characterized by deposits of IgA as either the dominant or codominant immunoglobulin (1,2). Proteinuria is regarded as a risk factor for an unfavorable renal prognosis and the reduction of proteinuria is considered an important therapeutic goal in clinical practice (3–5). In addition to proteinuria, active renal lesions, such as cellular crescent formation, diffuse mesangial proliferation and interstitial inflammatory infiltration have been identified to result in a rapid rate of deterioration and lower kidney survival (6).

At present, the optimal immunosuppressive strategy for IgAN treatment, particularly the duration of treatment, remains unclear. Treatment with glucocorticoids alone or combined therapy with glucocorticoids and other immunosuppressive agents have shown beneficial effects by reducing proteinuria and improving renal function (7,8). According to the guidelines ‘Kidney Disease: Improving Global Outcomes’ (KDIGO) (9), which are based on the results of previous studies (7,10,11), patients with persistent proteinuria of >1 g/day following 6 months of treatment with renin-angiotensin system (RAS) inhibitors are suggested to receive a 6-month course of corticosteroid therapy. However, certain issues require clarification, including whether immunomodulatory treatment should be continued or withdrawn in patients with proteinuria of <1 g/day following 6 months of corticosteroid treatment, and whether the complete resolution of proteinuria is equivalent to the disappearance of renal active lesions. Furthermore, the effectiveness of immunosuppressive regimens other than glucocorticoid monotherapy for this heterogeneous disease require investigation

In the present study, the effects of an individualized, low-dose multi-drug immunosuppressive regimen on IgAN treatment were retrospectively evaluated and a preliminary investigation of the duration of immunosuppressive treatment for IgAN based on repeat renal biopsies was conducted.

## Materials and methods

### Data collection

Clinical data of 17 patients diagnosed with primary IgAN by biopsy, including 11 males and 6 females with a mean age of 30.5 years (range, 14–61 years) were collected from the medical records at The Affiliated Hospital of Guangdong Medical College (Zhangjiang, China). All patients were notified and agreed for their clinical data to be used in this study. The institutional review board of the Affiliated Hospital of Guangdong Medical College approved this study and waived the requirement for patient consent.

### Therapeutic intervention

None of the patients received steroids or other immunosuppressive agents prior to the first renal biopsy. As shown in Table I, following the first biopsy, 16 patients were regularly treated with low-dose prednisone (PDN) alone or in combination with one or two other low-dose immunosuppressants. One patient (patient no. 2) discontinued immunosuppressive therapy from months 9 to 28. Generally, the immunosuppressive regimen was individualized according to the patient’s clinical and pathological presentation at the first biopsy. Briefly, in severe cases of proteinuria and/or the marked presence of active renal lesions the high dose immunosuppressants were administered. The initial dose of PDN was ~0.5 mg/(kg·day) for 8 weeks followed by a 10% reduction of the original dose in 1 month and gradual tapering to maintain a dose of 5–10 mg/day. *Tripterygium wilfordii* Hook F (TwHF) was administered orally at an initial dose of ~1.0 mg/(kg·day) in three divided doses for ~6 months and gradually tapered to 20–30 mg/day as the maintenance therapy. Azathioprine (AZA) was administered at a single dose of 1–2 mg/(kg·day) for ~6 months and gradually tapered to 25–50 mg/day as the maintenance therapy. Intravenous cyclophosphamide (CTX) was administered at 0.8–1.0 g per month for 3 months, followed by 1–2 mg/(kg·day) AZA for an additional 3 months and then gradually tapered to 25–50 mg/day. All patients received angiotensin-converting-enzyme inhibitor (ACEI) and/or angiotensin II receptor blocker (ARB) as part of their anti-hypertensive regimen or their basic therapy. Six patients (patient nos. 4, 6, 9, 11, 12 and 14) received lipid-lowering agents due to the presence of hyperlipidemia and 8 patients (patient nos. 1, 2, 3, 4, 5, 7, 10 and 11) received allopurinol due to hyperuricemia.

### Follow-up

Sixteen patients underwent regular follow-ups at the outpatient clinic approximately once a month until they received the second biopsy. Patient no. 2 discontinued follow-up from months 9 to 28 but continued from months 28 to 50.5. During the follow-up, the clinical symptoms, drug consumption and possible treatment complications of the patients were evaluated. Blood pressure was monitored and standard body examinations were performed. Laboratory parameters, including serum levels of creatinine, uric acid, cholesterol and albumin, as well as results of routine blood tests were recorded. Every month, urinary protein was semi-quantified. The 24-h urinary protein excretion level was measured at the first and second renal biopsies. The glomerular filtration rate (GFR) was estimated using the Modification of Diet in Renal Disease study (MDRD) formula: [eGFR (ml/min/1.73m^2^) = 186 * Scr (mg/dl) − 1.154 * age (years) − 0.203 * (0.742 femal].

### Renal biopsies and pathological diagnosis

All patients underwent renal biopsies twice with an interval time of 16.2±9.3 months (range, 9.0–50.5 months) under ultrasound guidance. Following hematoxylin and eosin, Masson’s trichrome, silver methenamine, periodic acid-Schiff and immunofluorescent staining, the renal specimens were assessed by one renal pathologist and one clinical nephrologist who were blinded to the clinical information. In immunofluorescent staining, the deposition of IgA, IgG, IgM and complement C3 was semi-quantified as follows: Negative, (−); minimal in intensity, (±); slight in intensity, (1+); moderate in intensity, (2+); marked in intensity, (3+); and marked in intensity and extent, (4+). These semi-quantified results were converted into scores as follows: 0, (−); 0.5, (±); 1, (1+); 2, (2+); 3, (3+); and 4 (4+), respectively. The activity and chronicity indices were evaluated as previously described (12). Briefly, the activity index was first graded for mesangial proliferation (grades 0–3: Normal, 0; slight, 1; moderate, 2; and severe, 3), interstitial inflammatory cell infiltration (grades 0–3: None, 0; 1–20%, 1; 21–50%, 2; and >50%, 3) and cellular crescent formation (grades 0–3 according to the percentage of glomeruli involved in crescents: None, 0; 1–20%, 1; 21–50%, 2; and >50%, 3). The sum of these scores was subsequently computed (maximum of 9). The chronicity index was first graded for the percentage of glomeruli exhibiting fibrous crescents (grades 0–3: None, 0; 1–20%, 1; 21–50%, 2; and >50%, 3), the percentage of glomeruli exhibiting segmental or global sclerosis (grades 0–3: None, 0; 1–20%, 1; 21–50%, 2; and >50%, 3), and the degrees of tubular atrophy (on a scale of 0–3) and interstitial fibrosis (on a scale of 0–3). The sum of these scores was subsequently computed (maximum of 12).

### Statistical analysis

Statistical tests were performed with SPSS software, version 16.0 for Windows (SPSS Inc., Chicago, IL, USA). Normally distributed variables were assessed using the paired-samples t-test. Nonparametric variables and non-normally distributed variables were assessed by Wilcoxon signed rank test. P<0.05 was considered to indicate a statistically significant difference.

## Results

### General data

The regimen was well tolerated and no severe adverse events were observed. Blood pressure in all patients was controlled to the standard of ~130/80 mmHg during the course of follow-up. According to renal pathological diagnosis of the second biopsy, five patients (patient nos. 5, 10, 14, 15 and 17) were able to discontinue immunosuppressive treatment as substantially no active renal lesions were observed, while the remaining patients continued the regimen with a very low dose of immunosuppressants due to existing residual active renal lesions.

### Clinical data

**Figure 1:**
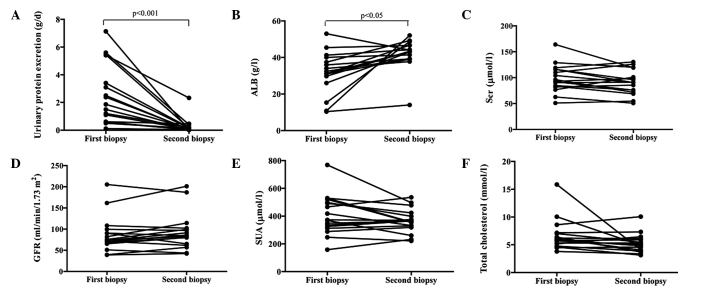
Comparison of the clinical and pathological data of 17 patients with IgAN at the first and second renal biopsies. (A) Urinary protein excretion decreased significantly from 2.53±2.17 g/day at the first biopsy to 0.26±0.55 g/day at the second biopsy (P<0.001); (B) ALB increased from 31.54±11.34 g/l at the first biopsy to 42.06±8.24 g/l at the second biopsy (P<0.05); (C) Scr decreased from 98.29±26.51 μmol/l at the first biopsy to 91.00±23.54 μmol/l at the second biopsy (P>0.05); and (D) GFR increased from 86.14±41.77 ml/min/1.73 m^2^ at the first biopsy to 92.62±43.13 ml/min/1.73 m^2^ at the second biopsy (P>0.05). GFR was calculated using the modification of diet in renal disease formula. (E) SUA and (F) total cholesterol levels were not significantly different between the two biopsies though both levels were reduced at the second biopsy in comparison with those at the first biopsy. IgAN, immunoglobulin A nephropathy; ALB, serum albumin; Scr, mean serum creatinine; GFR, glomerular filtration rate; SUA, serum uric acid.

Semi-quantified analysis showed that the urinary protein levels in all patients decreased gradually to normal levels from months 2 to 7 (data not shown). The mean 24-h protein excretion levels markedly declined from 2.53±2.17 g/day at the first biopsy to 0.26±0.55 g/day at the second biopsy (patient no. 2 was 2.33 g/day) (P<0.001; Fig. 1A). Correspondingly, the serum albumin levels increased significantly from 31.5±11.3 to 42.1±8.2 g/l (P<0.05; Fig. 1B). The serum creatinine levels decreased from 98.3±26.5 to 91.0±23.5 μmol/l (Fig. 1C), while the GFR increased from 86.1±41.8 to 92.6±43.1 ml/min/1.73 m^2^ (Fig. 1D); however, there were no significant differences in these levels between the two biopsies. Additionally, serum uric acid and total cholesterol levels were not significantly different between the two biopsies (Fig. 1E and F).

Notably, proteinuria decreased gradually to normal level at 5 months in patient no. 2 who received the combined therapy with a low-dose of PDN, TwHF and AZA. The patient discontinued immunosuppressive treatment at 9 months and proteinuria relapsed severely at 28 months. Although immunosuppressants were administered again following relapse, proteinuria was not alleviated until the second biopsy at 50.5 months.

### Pathological data

**Figure 2:**
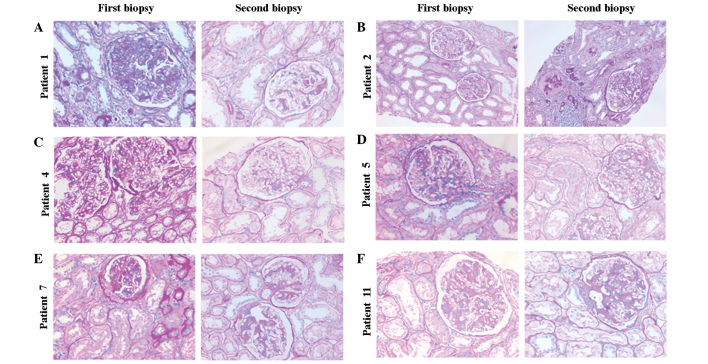
Representative histological sections following immunomodulatory therapy in immunoglobulin A nephropathy. All sections were stained with periodic acid-Schiff. (A) In patient 1, at the first renal biopsy moderate diffuse mesangial cell proliferation, increased matrix deposition, adhesion to the Bowman’s capsule and interstitial mononuclear cell infiltration were observed. At the second renal biopsy, slight mesangial cell proliferation and matrix expansion, but no adhesion to the Bowman’s capsule or interstitial mononuclear cell infiltration were observed. (B) In patient 2, compared with that at the first biopsy, segmental glomerulosclerosis, interstitial infiltration, tubular atrophy and interstitial fibrosis were observed in the second renal biopsy. The histopathological features had deteriorated at the second renal biopsy (magnification, ×200). (C,D,F) In patients 4, 5 and 11, the mesangial proliferation and matrix increases observed in the first biopsy were ameliorated at the second renal biopsy. No major changes of tubulointerstitial lesions were visible (magnification, ×400). (E) In patient 7, at the first renal biopsy, glomerular cell proliferation, matrix expansion, focal adhesion to the Bowman’s capsule, tubular atrophy and interstitial fibrosis were observed. At the second renal biopsy, mild mesangial cell proliferation and adhesion to the Bowman’s capsule were detected. (A,C–F) Histopathological changes were improved at the second renal biopsy (magnification, ×400).

The intensity of glomerular IgA and C3 deposits was dramatically decreased at the second biopsy compared with that at the first biopsy (P=0.004 and P=0.011, respectively); however, glomerular IgA and/or C3 deposits remained present in the majority of patients at the second biopsy (Table II). The intensity of other immunoglobulin deposition was not significantly different between the two biopsies (data not shown). The acute lesions, such as diffuse mesangial proliferation, cellular crescent formation or interstitial mononuclear cell infiltrates showed marked amelioration in the majority of patients at the second biopsy compared with that at the first (Fig. 2). In addition, chronic pathological damage, including glomerular capsular adhesion, segmental glomerular sclerosis, global sclerosis and the degree of interstitial fibrosis, did not show progression in the majority of patients (Fig. 2). As indicated by the results in Table II, the activity index decreased significantly from 3.18±1.33 to 2.47±0.80 (P<0.05), while the chronicity index remained unchanged (2.59±2.00 versus 2.76±1.89). Moreover, although the score of acute lesions decreased, residual renal active lesions were identified in 12 patients who had almost complete remission of proteinuria at the second renal biopsy. Histopathology was aggravated severely in patient no. 2, observed as severe glomerular sclerosis and interstitial fibrosis (Fig. 2B).

## Discussion

The benefits of corticosteroid monotherapy for the treatment of IgAN have been elucidated in several studies (7,13,14) and suggested in the KDIGO guidelines. However, an additional randomly controlled trial conducted in Japan showed that corticosteroid monotherapy was not beneficial for protecting kidney function (15). AZA is not suggested for the treatment of IgAN according to the KDIGO guidelines; however, the addition of AZA to corticosteroids has been shown to provide further benefits in the reduction of proteinuria and the stabilization of renal function in patients with IgAN, particularly in those who did not respond to corticosteroid monotherapy (16). Notably, Shima *et al* showed that glomerular IgA deposits disappeared following combined therapy with corticosteroids and AZA in children with IgAN showing diffuse mesangial proliferation (17). TwHF, an extract from a traditional Chinese medicine, has been widely used to treat autoimmune and inflammatory diseases, including lupus nephritis and rheumatoid arthritis (18–20). A previous study showed that Chinese patients with IgAN benefited from TwHF treatment (21). In conclusion, the optimal treatment strategy for IgAN remains controversial, despite the KIDIGO guidelines.

In the past few years, prior to the publication of the KDIGO guidelines for IgAN treatment, we applied an individualized, low-dose and multi-drug immunosuppressive regimen for the treatment of IgAN based on clinical and pathological patterns, which was inspired from the idea of multi-target therapy for lupus nephritis (22). All patients in the present retrospective study received relatively low-dose PDN treatment in order to avoid the obvious side-effects of high-dose steroids. However, to compensate for the insufficient immunosuppressive strength of low-dose PDN, AZA or a short course of CTX was administered to those patients with more severe proteinuria and/or active pathological changes. The dosage was flexibly adjusted according to the condition of the patient. In particular, the traditional Chinese medicine, TwHF, was also added to the two immunosuppressants in the majority of patients, which formed a triple immunosuppressive regimen. Notably, it was observed that this immunosuppressive regimen significantly decreased proteinuria, increased serum albumin levels and stabilized renal function. Complete remission of proteinuria was achieved in the majority of patients. However, it may be suggested that basic therapy, such as ACEI and/or ARB usage and blood pressure control may have also contributed to the reduction in proteinuria. However, according to previous studies, basic RAS inhibition fails to achieve complete remission of proteinuria in the majority of patients with IgAN (23,24), which suggests that immunosuppressive treatment may be important for proteinuria remission in the patients in the present study. Furthermore, a decline in the renal pathological activity index indicated that renal inflammation was controlled at the second biopsy. The chronicity index did not increase in the majority of patients, suggesting that no additional active renal lesions developed into chronic destruction. It has been suggested that the intensity of glomerular IgA and C3 deposition may be associated with IgAN progression (25,26) and the disappearance of IgA indicates a better prognosis (17). In the present study, the quantities of IgA and C3 deposits were significantly decreased, indicating that the remission of proteinuria and improvement of renal pathological lesions may be due to the minimizing of the abnormal immune inflammatory response by the combined immunosuppressive strategy, and may be independent to the basic therapy. This supports the positive therapeutic effects of this immunosuppressive regimen on IgAN. In addition, the side-effects were mild and patients successfully tolerated the immunosuppressive regimen.

According to the KDIGO guidelines, the duration of corticosteroid treatment is 6 months in patients with IgAN and proteinuria of >1 g/day. However, the present study indicated that although proteinuria was markedly decreased (as shown in Fig. 1A), the deposits of IgA and C3 and renal active lesions remained in the glomerulus following combined immunosuppressive treatment for ~1 year in the majority of patients. This indicated that complete remission of proteinuria did not conform to the disappearance of histopathological activity. Thus, a 6-month course of immunosuppressive therapy may not be enough to completely control renal active lesions and a longer course of treatment may be required for certain patients with IgAN. Moreover, in patient no. 2, proteinuria relapsed following the withdrawal of immunosuppressants. The patient had not achieved complete remission of proteinuria and chronic histopathological changes with significant deterioration were observed at the second renal biopsy, which may have resulted from failure to continue treatment. Therefore, remission of proteinuria may not be the only decisive factor for withdrawing immunosuppressants in certain patients with IgAN and additional factors, such as renal pathology should be considered (12,27).

It is widely considered that proteinuria of <1 g/day indicates a favorable prognosis (3) and such patients require no specific treatment, but should be kept under periodic review (28,29). However, Usui *et al* (30) demonstrated that certain patients with mild proteinuria (<0.5 g/day) progress to dialysis. In the present study, five patients with an initial proteinuria of <1 g/day presented marked active renal lesions at the first biopsy and were administered a very low-dose of the multi-drug immunosuppressive regimen for ~1 year. During follow-up, the patients achieved complete remission of proteinuria and their renal pathological lesions improved. This supports the idea that immunosuppressive therapy will bring additional benefits for those patients with IgAN whose urinary protein is <1 g/day, but show existing active renal lesions.

It should be acknowledged that there are limitations in the present study, including the lack of a control group, a small sample size, a retrospective study design and the relatively short follow-up period. Therefore, the optimal drug-combined regimen and duration of immunosuppressive therapy in patients with IgAN requires further study.

In conclusion, the individualized low-dose multi-drug immunosuppressive regimen significantly minimized proteinuria, stabilized renal function and alleviated histological lesions without inducing overt adverse effects in patients with IgAN in the short-term follow-up. However, optimal treatment strategies require further investigation. Notably, the results also indicated that additional factors to proteinuria, particularly renal pathological alterations, should be considered when withdrawing immunosuppressant therapy from IgAN treatment. The duration of immunosuppressive therapy on IgAN requires further study.

## Figures and Tables

**Table I:** Baseline characteristics of 17 patients with IgA nephropathy who underwent a repeat renal biopsy.

Patient	Gender	Age at first biopsy (years)	Interval (months)	RAS inhibitors	Immunosuppressive treament
1	F	28	12	Yes	PDN + TwHF + AZA
2	M	21	50.5	Yes	PDN + TwHF +AZA
3	M	18	9	Yes	PDN + TwHF + CTX → AZA
4	M	19	15	Yes	PDN + TwHF + AZA
5	M	14	15	Yes	PDN + TwHF + AZA
6	F	59	21	Yes	PDN + TwHF
7	M	22	17.5	Yes	PDN + TwHF + AZA
8	M	61	12.5	Yes	PDN + TwHF + AZA
9	F	31	14	Yes	PDN + TwHF + AZA
10	F	39	14	Yes	PDN + TwHF + AZA
11	M	26	12.5	Yes	PDN + TwHF + AZA
12	M	35	15	Yes	PDN
13	M	14	11	Yes	PDN + AZA
14	F	32	16.6	Yes	PDN + TwHF + CTX → AZA
15	M	26	17	Yes	PDN + TwHF + AZA
16	M	55	12	Yes	PDN + AZA
17	F	18	11.5	Yes	PDN + TwHF

IgA, immunoglobulin A; RAS, renin-angiotensin system; PDN, prednisone; TwHF, *Tripterygium wilfordii* Hook F; AZA, azathioprine; and CTX, cyclophosphamide.

**Table II:** Histological data of 17 patients with IgAN who underwent a repeat renal biopsy.

		Intensity of deposits		Index of renal pathological lesions		
			
Patient	Biopsies	IgA	C3	AS	CS	TS
1	First	3	3	5	4	9
	Second	2	2	4	2	6
2	First	2	0	2	0	2
	Second	2	0	3	6	9
3	First	1	4	4	5	9
	Second	1	1	2	3	5
4	First	1	0	2	1	3
	Second	0.5	0	1	0	1
5	First	3	0.5	2	0	2
	Second	2	0.5	3	1	4
6	First	4	4	3	5	8
	Second	4	1	2	6	8
7	First	3	2	3	4	7
	Second	3	2	3	3	7
8	First	3	2	6	3	9
	Second	3	2	2	3	5
9	First	4	2	2	0	2
	Second	2	1	2	4	6
10	First	3	2	5	5	10
	Second	2	2	4	6	10
11	First	4	3	5	1	6
	Second	2	2	3	1	4
12	First	2	0.5	2	1	3
	Second	2	0	2	2	4
13	First	4	3	3	3	6
	Second	3	3	2	2	4
14	First	2	0.5	2	6	8
	Second	1	0	2	2	4
15	First	3	2	2	2	4
	Second	3	2	2	1	3
16	First	3	0	3	3	6
	Second	1	0	3	4	7
17	First	3	3	3	1	4
	Second	2	2	2	1	3

IgAN, immunoglobulin A nephropathy; AS, activity index score; CS, chronicity index score; TS, total score. The intensity of deposits detected by immunofluorescent microscopy was graded semi-quantitatively on a scale from 0 to 4: Negative, 0; minimal in intensity, 0.5; slight in intensity, 1; moderate in intensity, 2; marked in intensity, 3; and marked in intensity and extent, 4.
